# Research progress of spike protein mutation of SARS-CoV-2 mutant strain and antibody development

**DOI:** 10.3389/fimmu.2024.1407149

**Published:** 2024-11-18

**Authors:** Xinkang Huan, Jiuyu Zhan, Hongwei Gao

**Affiliations:** School of Life Science, Ludong University, Yantai, Shandong, China

**Keywords:** COVID-19, SARS-CoV-2, variants, inevitability, antibody

## Abstract

The coronavirus disease 2019 (COVID-19) is a respiratory disease with a very high infectious rate caused by the Severe Acute Respiratory Syndrome Coronavirus-2(SARS-CoV-2). Because SARS-CoV-2 is easy to mutate, the continuous emergence of SARS-CoV-2 variant strains not only enhances the infectivity of the SARS-CoV-2 but also brings great obstacles to the treatment of COVID-19. Neutralizing antibodies have achieved good results in the clinical application of the novel coronavirus pneumonia, which can be used for pre-infection protection and treatment of novel coronavirus patients. This review makes a detailed introduction to the mutation characteristics of SARS-CoV-2, focusing on the molecular mechanism of mutation affecting the infectivity of SARS-CoV-2, and the impact of mutation on monoclonal antibody therapy, providing scientific reference for the prevention of SARS-CoV-2 variant strains and the research and development of antibody drugs.

## 1 Introduction

Following SARS-CoV (Severe Acute Respiratory Syndrome Coronavirus) and MERS (Middle East Respiratory Syndrome Coronavirus), SARS-CoV-2 is the third coronavirus that can cause severe respiratory disease in humans (1). SARS-CoV-2 has spread widely and lasted for a long time, and the coronavirus disease 2019(COVID-19) caused by SARS-CoV-2 has put enormous pressure on global healthcare systems and seriously threatened the health of people around the world. As of 1 September 2024, the cumulative total Number of COVID-19 cases reported to WHO has reached 776 million, with 7.06 million cumulative deaths, and is growing (2). At the same time, various vaccines, monoclonal antibodies, and small molecule inhibitors against SARS-CoV-2 have been developed, dozens of monoclonal antibodies have been approved for the treatment of SARS-CoV-2, and hundreds of monoclonal antibodies are in the clinical research stage. The development of these drugs provides a powerful weapon to contain the SARS-CoV-2 and prevent the COVID-19 pandemic.

However, with the pandemic of COVID-19 pneumonia, there have been many variants of SARS-CoV-2 around the world, especially mutations in spike proteins, resulting in enhanced transmission capacity of the SARS-CoV-2 and increased immune escape ability. According to the World Health Organization’s classification of the safety risk level of novel coronavirus variants, the definition of variants of concern (VOC), and variants of interest (VOI). The pathogenicity and virulence of VOC are far more than other strains, and they have a significant impact on the spread of the epidemic, the rate of severe illness, mortality, and drug resistance.

In the face of these problems, we need to understand these variants comprehensively, and according to the characteristics of the variants, we can conclude the antibody drugs that can effectively control these variants. In this review, we summarized the evolution history of SARS-CoV-2 mutation, the molecular characteristics of the mutation (mainly concentrated in the RBD region of the S protein), and systematically evaluated the efficacy of monoclonal antibodies against the mutation. In this way, more targeted treatments for the SARS-CoV-2 variant strains will provide a theoretical basis for modifying and optimizing monoclonal antibodies.

## 2 Structural characteristics and variation history of SARS-CoV-2

### 2.1 Structure and genomics of SARS-CoV-2

**Figure 1:**
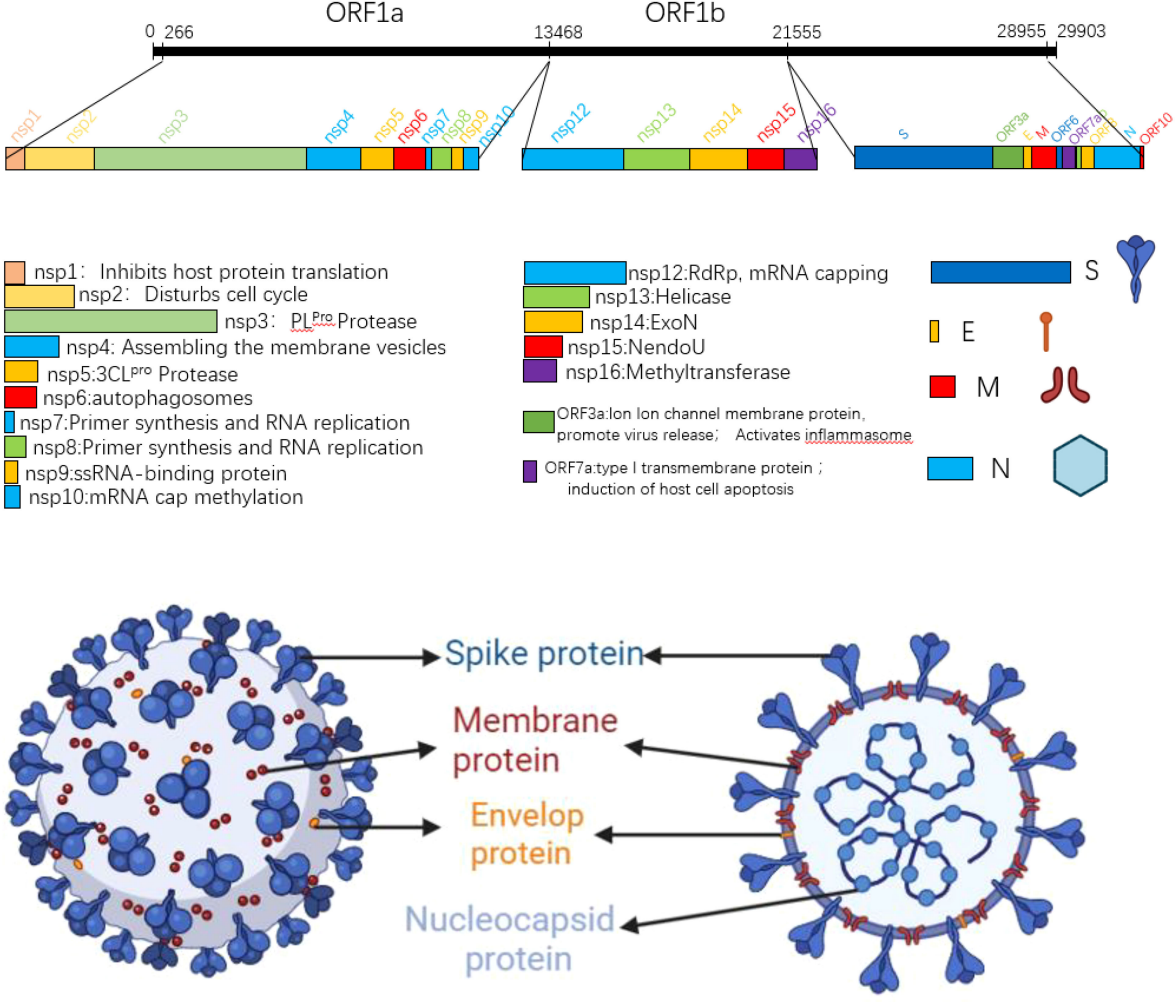
SARS-CoV-2 gene map and planar structure of virus particles.

The SARS-CoV-2 genome is approximately 29.8kb in size and contains 14 open reading frames (ORFs) that encode 27 proteins. From the 5 ‘end to the 3’ end of the genome, two polymeric protein precursors pp1a and pp1ab, four structural proteins (spike protein (S), envelope protein (E), membrane protein (M), nucleocapsid protein (N)), and a series of coding auxiliary proteins are encoded (3, 4). In the process of viral proliferation, the polymeric precursors pp1a and pp1ab are cut by viral protease into 16 non-structural proteins (NSPs), which play a very important role in viral genome replication and transcription. The translated structural proteins are an essential part of the virus structure, which are mainly involved in the assembly of virions and the suppression of cellular immune response. In addition to ORF3a and ORF7a, the remaining helper proteins regulate viral infection (5). SARS-CoV-2 is A righteous single-stranded RNA virus, and the GC content in its genome is particularly low, only 38%. Since cytosine and uracil were previously composed of three hydrogen bonds, the structure is more stable than A-T, and all the low GC content also leads to the unstable transcription and translation of SARS-CoV-2. It is easy to replace a single amino acid, leading to mutations (5). The SARS-CoV-2 virus particle is round or oval, the particle size is about 80-120nm, and it belongs to Betacoronavirus (6). Its surface is covered by a lipid bilayer envelope, on which many rod-like homologous trimer S protein structures are scattered outward, thus giving the virus a crown shape, hence the name coronavirus (7). Inside the envelope is the nucleocapsid structure; the nucleocapsid is a helically symmetric capsid protein, +ssRNA complex. **Figure 1** shows the SARS-CoV-2 genome structure and virus model (8, 9).

### 2.2 History of the evolution of the SARS-CoV-2 variant

There are many reasons for the emergence of SARS-CoV-2 variants, which can be roughly divided into three categories: the most fundamental is the instability of single-stranded RNA, the limited error-correcting ability of RNA polymerase in its replication process, and the acceleration of self-evolution caused by SARS-CoV-2 mutations (10); The second is the instability of the spike protein domain (11). Compared with other domains, the spike protein is relatively loose, and the replacement of amino acids in the spike protein domain can easily adapt to the new structure; Finally, there is the recombination between different variants of the coronavirus (12). In order to effectively detect SARS-CoV-2 variants, WHO uses the Greek alphabet to classify viruses, but there are also a number of other naming systems, including GASAID, PANGO, and Nextstrain, in order to distinguish those with high health risks, WHO has divided them into two main categories: variants of concern (VOC) and variants of interest (VOI). Alpha (B.1.1.7), Beta (B.1.351), Gamma (P.1), Delta (B.1.617.2), and Omicron (B.1.1.529) are considered previously popular VOCS, while VOI includes Lambda (C.37) and Mu (B.1.621).

**Figure 2:**
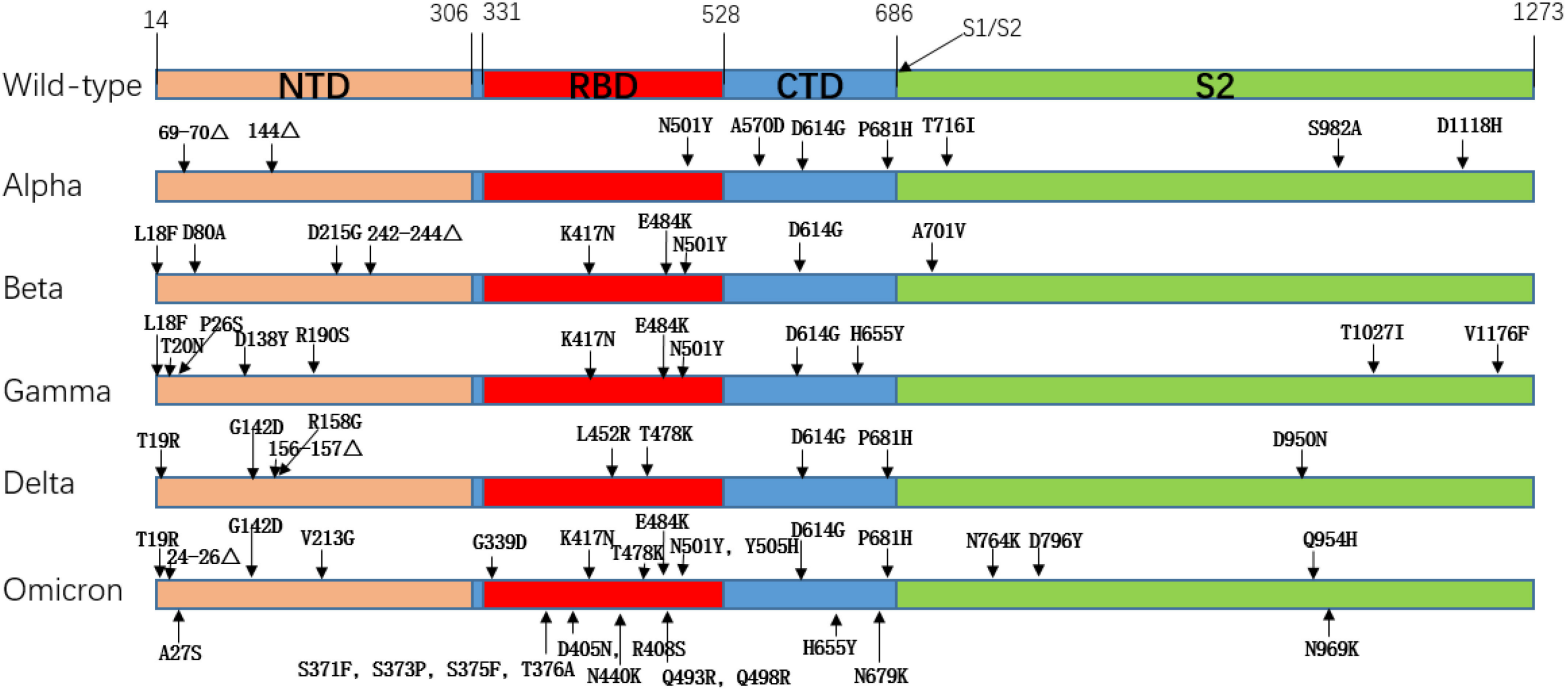
Amino acid mutation of spike protein in SARS-CoV-2 mutant strain.

The earliest globally circulating mutant was the D614G mutation, which enhanced the cleavage ability of Flynn protease at the S1/S2 junction so that the SARS-CoV-2 variant increased its infectivity and replication while retaining similar virulence to the original strain (13). The rapid spread of the SARS-CoV-2Alpha variant (B.1.1.7), which first emerged in the South East of the United Kingdom in September 2020 and led to a UK-wide increase in COVID-19 incidence within two months and traces of the variant in 114 countries within six months, illustrates the superiority of Alpha in terms of transmission (14, 15). The Alpha variant has nine mutations on the S protein, the most biologically representative of which is the N501Y replacement, where Tyr replaces the Asn at 501, and the mutation occurs in the SARS-CoV-2 receptor binding domain (RBD), which improves the affinity with the ACE2 receptor (16). The Beta (B.1.351) variant was first detected in South Africa in October 2020, with a total of 9 mutations on the S protein, among which K417N, E484K, and N501Y are mutations occurring on RBD, which promote the immune recognition of the virus to escape host cells and enhance its affinity with ACE2 (17). Specifically, for the E484K mutation, the researchers evaluated the neutralization effect of several antibodies against the E484K mutation. They found that the neutralization effect of some monoclonal antibodies was reduced, and some antibodies targeting the n-terminal domain were ineffective against Beta variants. It is speculated that the Beta mutation leads to significant structural changes in the N-terminal domain (18). The Gamma (P.1) variant was detected in Brazil and Japan in November of the same year, and the number of mutations in the S protein of the Gamma variant increased to 12 mutations. The mutation of the Gamma variant included mutations of several other variants, including K417N, N501Y, D614G, etc. The transmissibility of the Gamma variant is estimated to be 1.7 to 2.4 times that of the original strain (19, 20). Delta (B.1.617.2) was detected in India almost simultaneously with the Bata mutation in October 2020. Still, in June 2021, the Delta variant quickly swept the world and became dominant globally. This was mainly related to the newly added mutation of Delta. The mutant strain of Delta had a total of 9 mutations in the S protein, among which the P681R mutation enhanced the cleavage of the S1/S2 subunit and promoted the binding of the S protein to the ACE2 receptor, resulting in a significant enhancement of the infectiveness and reproduction of Delta. This mutation would also lead to secondary infection (21). In addition, L452R and T478K mutations also appeared in Delta variant strains, and the appearance of these two mutations resulted in a decrease in the neutralizing efficacy of partially neutralizing antibodies or even a failure to play a neutralizing role (21). Compared with several other variants, the Delta variant has a broader spread range, more substantial virulence, and more vital immune escape ability, seriously endangers public health security. The last VOC, Omicron (B.1.529), was detected in November 2021, and Omicron was quickly defined as a VOC as soon as it was seen. According to the gene sequence, Omicron has more than 60 mutations, with 37 non-synonymous mutations in the S protein alone and 15 in the RBD region (22). It is also the VOC with the most mutations to date. We observed the mutation of Omicron and found that the mutant inherited several previous VOCs mutations, such as T478K, N501Y, D614G, etc. These mutations are responsible for the high affinity of Omicron to ACE2 (23). A high number of mutations means the loss of molecular targets. Many SARS-CoV-2 monoclonal antibodies target the RBD region, while Omicron has up to 15 mutations in the RBD region alone, which means that monoclonal antibodies have poor neutralization effect against Omicron, especially several sublines of Omicron. They showed remarkable immune evasion against monoclonal antibodies (24–27). This undoubtedly brings great difficulties to the development of antibody drugs. **Table 1** shows an overview of important VOC mutations of SARS-CoV-2, and **Figure 2** shows a summary of mutation locations of SARS-CoV-2 mutant strains.

**Table 1:** Summary of important VOC mutations of SARS-CoV-2.

Varian	Time and place of appearance	Number of S protein mutations	Mutations in the S protein	Transmission capacity	Neutralizing effect of antibody
Alpha(α)	September 2020, United Kingdom	9	69-70△,144△, N501Y,A570D,D614G, P681H,T716I,S982A, D1118H	Increased transmission(N501Y, P681H)	No impact on susceptibility to EUA mAb treatments
Beta(β)	October 2020, South Africa	9	L18F, D80A, D215G, 242-244△, K417N, E484K, N501Y, D614G, A701V	Increased transmission(N501Y, E484K, K417N)	Significantly decreased susceptibility to the Bamlanivimab and Etesevimab
Gammy(γ)	November 2020, Brazil, Japan	12	L18F, T20N, P26S, D138Y, R190S, K417T, E484K, N501Y, D614G, H655Y, T1027I, V1176F	Increased transmission(N501Y, E484K, K417N)	Significantly decreased susceptibility to the Bamlanivimab and Etesevimab
Delta(δ)	October 2020, India	9	T19R, G142D, 156-157△, R158G, L452R, T478K, D614G, P681R, D950N	Increased transmission(L452R, P681R)	Decreased sensitivity to antibodies targeting RBD and N-terminal domains
Omicron(o)	November 2021	37	T19R, 24-26△, A27S, G142D,V213G, G339D, S371F, S373P, S375F, T376A,D405N, R408S,K417N,N440K, S477N, T478K,E484A, Q493R,Q498R,N501Y, Y505H,D614G,H655Y, N679K,P681H,N764K, D796Y, Q954H,N969K	Significant increased transmission(N501Y, Q498R, H655Y, N697K, P681H)	Most antibodies lose neutralizing activity, bebtelovimab retains some neutralizing activity

### 2.3 The mutation of critical amino acids in the S protein of SARS-CoV-2 variant strains affected the transmission of the virus

ACE2 is widely distributed in human lungs, heart, liver, kidney, testicles, small intestine, pancreas, and other organs or tissues (28), SARS-CoV-2 invades cells mainly by binding to the ACE2 receptor on target cells through the receptor binding domain on the S protein (29). Therefore, the stronger the affinity between the receptor binding domain and the ACE2 receptor, the stronger the infectivity and transmissibility of SARS-CoV-2. Studies have shown that the binding interface between wild-type SARS-CoV-2 RBD and ACE2 receptor has 13 hydrogen bonds and 2 salt Bridges, and the equilibrium dissociation constant is 4.7nM (30).

#### 2.3.1 N501Y

**Figure 3:**
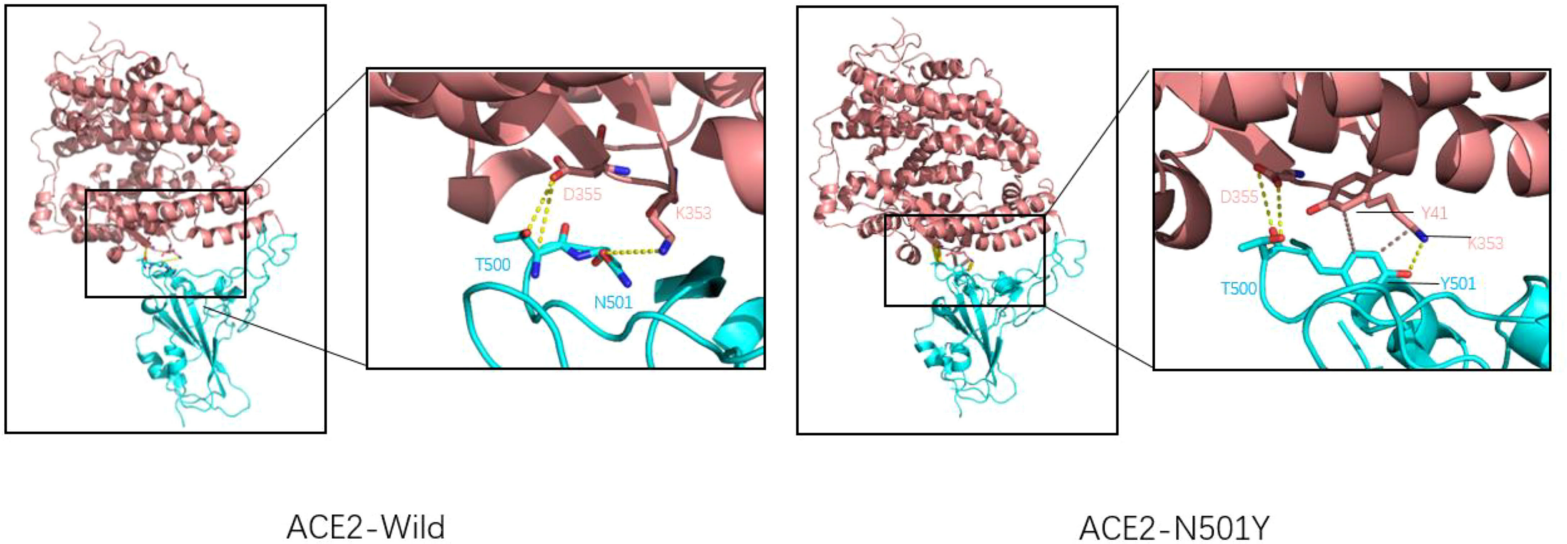
Wild-type COVID-19 RBD (left) and N501Y mutant COVID-19 RBD (right) binding to ACE2. ACE2 is shown in pink, RBD is shown in blue, Hydrogen bonds are shown by the dotted yellow line, and hydrophobic bonds by the dotted brown line. For natural spike proteins, Asn at N501 forms hydrogen bonds with Lys at 353 (2.71Å), and Thr500 forms two hydrogen bonds with amino acid residues Asp355 (2.61Å and 3.06Å). For the N501Y mutant RBD, Thr500 forms two hydrogen bonds with Asp355 (2.63Å and 2.63Å), Thr501 forms A hydrogen bond with Lys355 (2.93Å), A hydrophobic bond with Tyr41 (5.13 Å), and a hydrophobic bond with Lys353 (3.94 Å).

Researchers such as Neda Rostami use Rosetta docking protocol tracked the pattern of residues interactions for spike-ACE2 complex in both native and N501Y variants, which were found to form two hydrophobic bonds between the benzene ring of Tyr501 amino acid residue and tyr41 and lys355 amino acid residues of ACE2 receptor (**Figure 3**), which was not found in wild-type spike-ACE2 complex. Rosetta Energy showed that the spik-ACE2 complex with the N501Y mutation had a more stable structure. In addition, spike protein amino acid 501 mutates from asparagine to tyrosine, resulting in a better binding pocket at the binding interface. All the above phenomena indicate that N501Y mutation can lead to increased affinity of spike protein to ACE2 receptor (31). Xing Zhu and other researchers used luciferase detection to detect the pseudoviral infectivity of N501Y mutant protein and found that the relative luminous unit of N501Y mutant protein was twice as strong as that of unmutated spike protein, indicating that N501Y mutation resulted in enhanced infectivity (32).

#### 2.3.2 D614G

**Figure 4:**
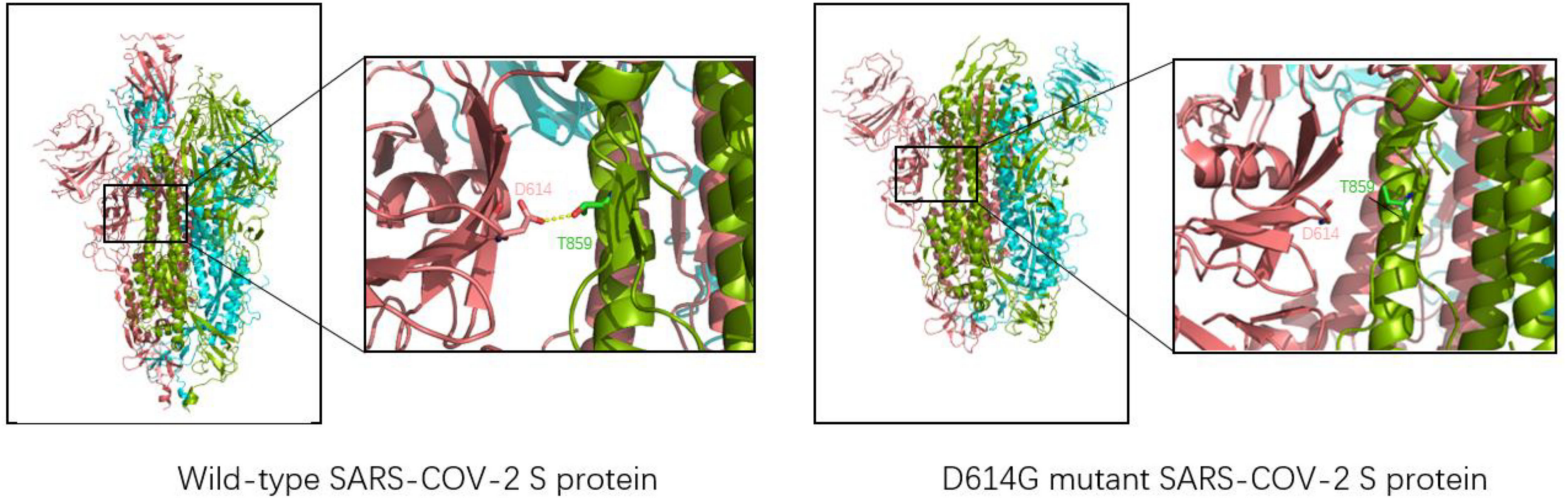
Three-dimensional structure of wild-type SARS-CoV-2 (left, structure reference PDB: 6VSB) and D614G mutant novel coronavirus (right, structure reference PDB:6XS6). The chains of each trimer S protein are shown in pink, blue, and green; Yellow dashed lines represent hydrogen bonds. For the natural spike protein, a hydrogen bond (2.7Å) is generated at Asn614 and Thr859 of the two chains, which is used to stabilize the stability of the trimer S protein. However, the distance between Asn614 and Thr859 in D614G-type S protein is too far to form hydrogen bond interaction, so it is unstable enough.

The D614G mutation, the first variant to cause a global pandemic, was first detected in Europe in January 2020 and rapidly spread worldwide in just one month, with significant implications for the spread of the virus (13). Jessica A. Pante and other researchers compared the replication dynamics of wild type and D614G SARS-CoV-2 on human lung epithelial Calu-3 cells. They found that the infectiveness of G614 virus was 1.2/2.4 and 1.9 times higher than that of D614 virus at 24/36/48h. In addition, the D614G SARS-CoV-2 showed less extracellular viral RNA while infecting cells. This suggests that the D614G mutation enhances the infectivity of SARS-CoV-2 (33). D614G mutation does not change the affinity or sensitivity of S protein binding to ACE2. Still, it changes the structure of the S protein itself so that SARS-CoV-2 reduces the shedding of the S1 subunit when invading, and the overall structure of the S protein with more functions is injected into cells, thus increasing the infectiousness of D614 SARS-CoV-2 (34). The whole-atom molecular dynamics simulation of the realistic D614G type mutation can make the novel coronavirus more easily show a single RBD upward state, making the critical epitopes of RBD more accessible, resulting in the spike protein more easily binding to the ACE2 receptor and increasing the infectivity of the novel coronavirus (35). As shown in **Figure 4**, D614 and Thr859 formed a hydrogen bond between two adjacent protomers in the original trimer spike protein to stabilize the stability of the trimer S protein. However, when the Asp at position 614 was mutated into Gly, the stability of the trimer was weakened, making the S protein not conducive to maintaining a symmetrical conformation. Thus, RBD showed an upward open state and was easier to combine with ACE2, which was consistent with previous studies (36).

#### 2.3.3 K417N/T

**Figure 5:**
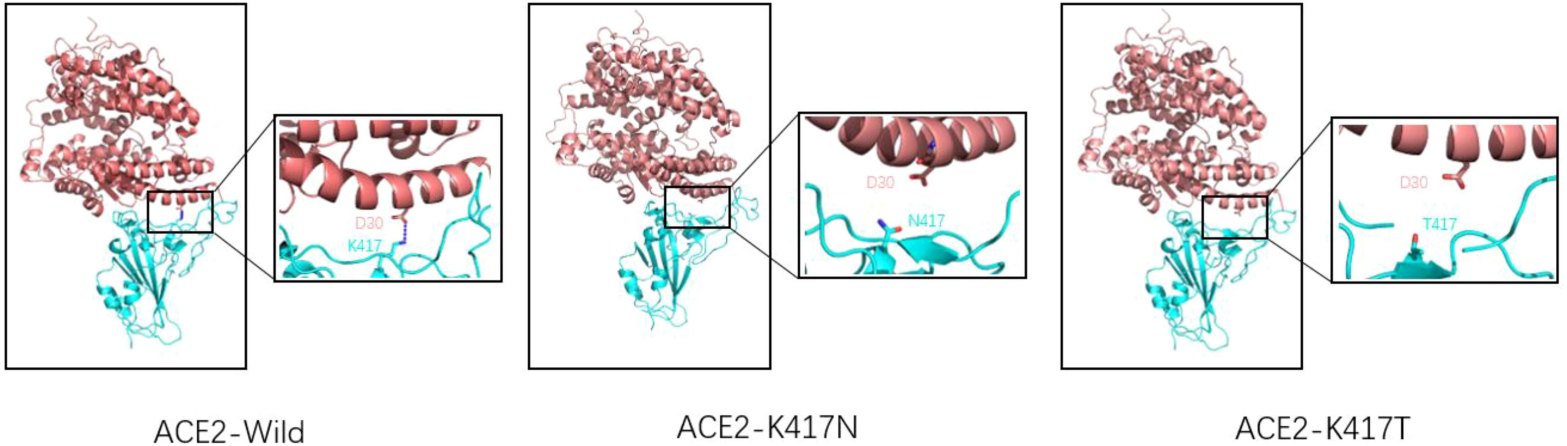
Wild-type SARS-CoV-2 RBD (left), K417N mutant RBD (middle), K417T mutant RBD (right), and ACE2 receptor complex model (reference structure PDB:6M0J) Pink is ACE2 structure, blue is RBD structure. A dashed blue line represents the salt bridge. Natural spike proteins form a salt bridge with D30(ACE2) at K417, the only salt bridge in the entire complex, and serve to stabilize the structure of the ACE2-RBD complex. However, K417N and K417T mutations lead to lysine mutation at position 417 to either asparagine or threonine, eliminating the only existing salt bridge.

K417N/T mutation occurred in Beta, Gamma, Delta, and Omicron mutant strains. K417N/T mutation was detected in South Africa for the first time and continued appearing in several subsequent VOCs. The production of K417N/T mutation has an essential impact on the transmission of the novel coronavirus (37). As for a single mutation of K417N/T, this mutation will lead to a decrease in affinity with ACE2, mainly because K417 will form a salt bridge with D30(ACE2). When Lys at 417 is replaced by Asp or threonine, the salt bridge between ACE2 and RBD will be eliminated (38, 39). (As shown in **Figure 5**) The molecular dynamics simulation of the salt bridge shows that the salt bridge is highly stable, and its existence time is much longer than that of ordinary salt Bridges (40). As the only salt bridge between RBD (wild type) and ACE2, K417-D30 plays a significant role in the stability of the complex. However, K417N/T mutations do not appear alone; in Beta, Gamma, and other VOCs, often accompanied by E484K, N501Y mutations occur, K417N/T, and the combination of these mutations often leads to an increase in affinity. According to the kinetic measurement of the mutant variant, it was found that the combination of K417N, E484K, and N501Y increased the affinity of RBD to ACE2 by 3.7 times, and the combination of K417T, E484K, and N501Y increased the affinity of RBD to ACE2 by 5.3 times (38). Subsequent experimental studies by Kim Y et al. also proved this point. The virus’s and host cells ‘ affinity was enhanced when the K417N mutation and E484K mutation were combined. Through more detailed surface charge analysis, it was found that the mutation of E484K from glutamic acid to lysine changed the electronegativity of amino acid residues here, thus generating a more stable salt bridge. To compensate for the loss of the salt bridge caused by the K417N mutation (37). The K417T mutation had results similar to those of the K417N mutation. When K417T was combined with E484K and N501Y mutations, it was found that a unique salt bridge was formed between Lys483 and Glu30 (ACE2), resulting in electrostatic interaction and greatly enhanced binding force (41). In conclusion, the appearance of the K417N/T mutation will lead to a decrease in the affinity between RBD and ACE2. Due to the diversity of mutations, the K417N/T mutation will be combined with any other mutation, and the mutation assembly will show a trend of increasing affinity.

#### 2.3.4 E484K

**Figure 6:**
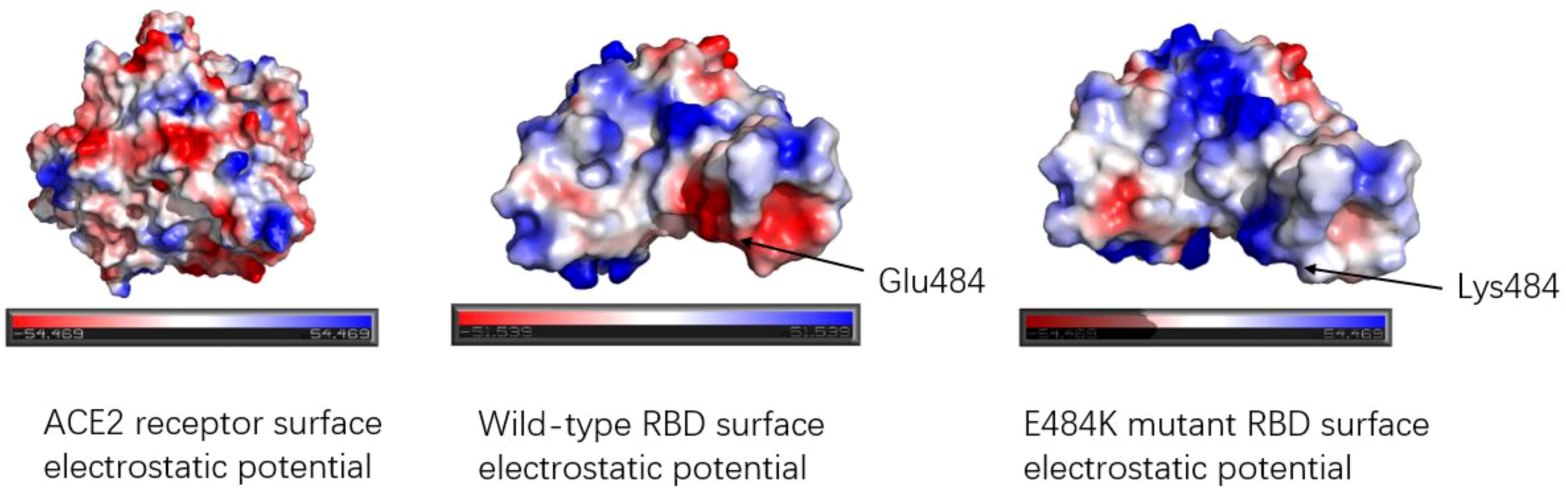
Electrostatic potential diagram of the binding interface between ACE2 receptor and RBD; Electrostatic potential diagram between wild-type RBD, E484K mutant RBD, and ACE2. The red color means the negative electrostatic potential, and the blue color indicates the positive electrostatic potential. The electrostatic potential of the ACE2 receptor binding interface showed negative electrostatic potential. The surface of RBD shows positive electrostatic potential as a whole. Glu484 of the wild type exhibited a strong negative electrostatic potential near the residue, while Glu484 mutated into Lys484, changed the electrostatic potential of amino acid 484th and surrounding amino acid residues, presenting a positive electrostatic potential, which was more favorable for binding with ACE2.

**Figure 7:**
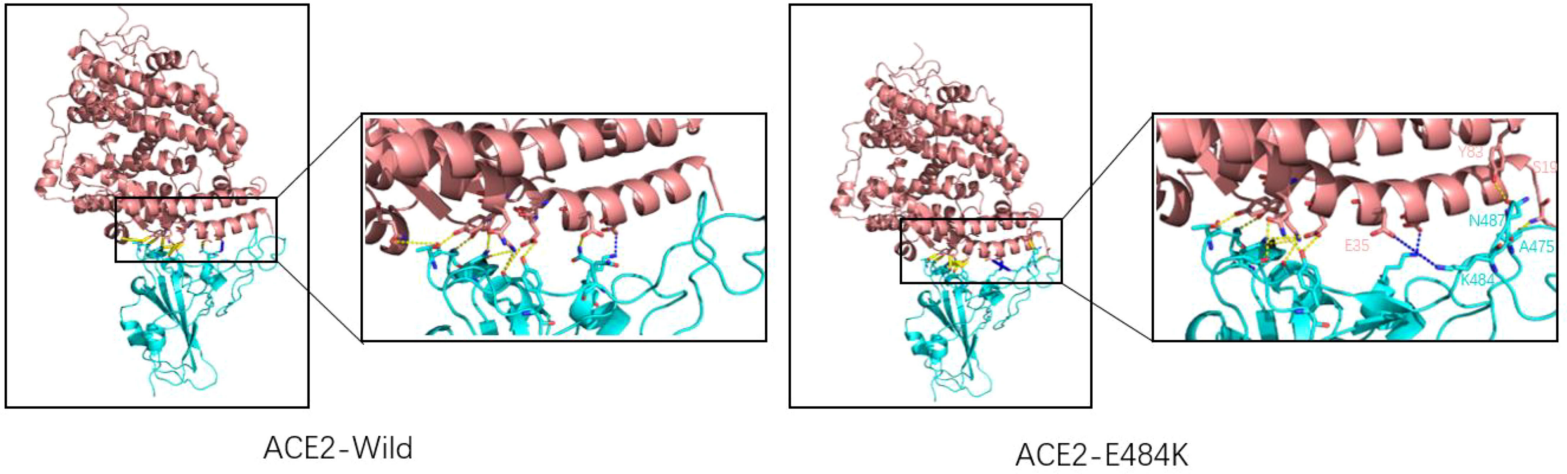
Wild-type SARS-CoV-2 RBD (left) and E484K mutant SARS-CoV-2 RBD (right) combine with ACE2. ACE2 is represented by pink, and RBD is represented by blue. The dashed yellow line shows the hydrogen bond and the blue line indicates the salt bridge. There is a salt bridge (K417-Glu30) for natural spike proteins, and hydrogen bonds are mostly clustered on the left side. The E484K mutation adds a salt bridge (Lys484-Glu35) to the original, with two more hydrogen bonds on the right side than the wild type (Asn487-Tyr83, Ser19-Ala475).

The E484K mutation in the RBM region was first detected in South Africa and was present in Beta, Gamma, Eta (B.1.525), Iota (B.1.526), and other variants. Studies have shown that the E484K mutation can increase the affinity of SARS-CoV-2 RBD to the ACE2 receptor. Weibu Wang et al. analyzed the electrostatic potential of the binding interface of the RBD-ACE2 complex and found that there were multiple charged residues on the ACE2 receptor, which exhibited negative electrostatic potential as a whole, and positive electrostatic potential as a whole on the binding interface of RBD, but strong negative electrostatic potential around Glu484 (RBD). This is undoubtedly not conducive to the binding of the ACE2-RBD complex. However, when Lys replaces Glu, the positive electrostatic potential of the RBD binding interface is increased, which will be more conducive to the binding of ACE2-RBD (42). **Figure 6** shows the surface electrostatic pattern of the ACE2 receptor binding interface with RBD. According to Chan et al., large buried surface area (BSA) facilitates the binding of RBD to ACE2 receptors (43). A large buried surface area (BSA) facilitates RBD binding to ACE2 receptors. At the same time, George Rucker, calculating the BSA of the E484K mutation in a 120nm molecular dynamics simulation, found that The E484K variant has a larger burial surface area than the original RBD and is, therefore, more conducive to the binding (44). The E484K mutation is located at the interface where RBD binds to ACE2, and mutations at this location tend to cause changes in interacting amino acids, as do mutations in E48K. Compared to wild-type SARS-CoV-2 RBD, the E484K mutation generates an additional salt bridge (Glu35-Lys484) that enhances binding affinity and infectivity (41). As shown in **Figure 7**, the mutation of E484K causes the number of salt Bridges to increase by one. Another primary reason for E484K’s affinity enhancement is that the mutation leads to a conformational rearrangement of amino acids around Lys484, resulting in an overall convergence of amino acid residues in the 489-494 region toward the ACE2 receptor. The structure comparison with the wild-type RBD-hACE2 complex showed that the R group of Tyr489 was close to Tyr83 of the ACE2 receptor and formed additional hydrogen bonds. In addition, additional hydrogen bond networks were formed between Tyr489-GLN24 (ACE2) and Asn487-Tyr83 (ACE2) (42). Mutual attraction of electrostatic potential, larger buried surface area, and additional salt bridge and hydrogen bond networks all explain the increased binding power of the mutant complex.

#### 2.3.5 L452R

The L452R mutation was discovered in the Delta strain in India in October 2020, and in just a few months, it quickly swept the world, becoming the most dangerous strain at the time. Leu452 is located at the binding interface of the RBD-ACE2 complex but is not directly involved in interacting with ACE2. Analysis of the RBD structure of wild-type novel coronavirus shows that Leu452 interacts with three hydrophobic amino acids, Leu492 and Phe490, to form hydrophobic plaques on the surface of RBD. At the same time, the L452R mutation mutates hydrophobic leucine into hydrophilic arginine, destroying the hydrophobic plaques on the surface of RBD. This results in increased affinity with ACE2 (45). In addition, the combination of L452R and P681R was observed in the Delta plant type. The hydrophobic amino acids Leu and Pro were replaced by hydrophilic Arg, indicating that the trend of virus evolution is to reduce the hydrophobicity of the binding interface and increase the hydrophilic interaction with ACE2, thus forming a more stable interaction network to increase the infectiability of the virus (46). Siddharth Sinha and other researchers also proved that L452R mutation can enhance the transmission of SARS-CoV-2. They used the MM/GBSA method to evaluate the binding free energy change between the L452R mutation and the ACE2 receptor. It was further demonstrated that L452R mutation can reduce the complex’s binding free energy and make it more stable (47). Molecular dynamics simulation and intermolecular affinity analysis showed that the L452R mutation resulted in RBD with a larger contact surface area and higher intermolecular interaction, which enhanced the affinity of the mutant for ACE2 (48). Another reason for the enhanced affinity caused by the L452R mutation is that the mutation promotes electrostatic complementarity at the ACE2-binding interface. Amino acid residue 452 is close to the negatively charged Glu35, Glu37, and Asp38 on the ACE2 receptor, and the substitution of L452R leads to increased electrostatic interaction at the RBD-ACE2-binding interface (49). The reason for the high infectivity of L452R mutation is not only that L452R mutation can increase the affinity between RBD-ACE2 but also that L452R mutation can increase the stability of this diagram protein and the fusion of the virus to promote the replication of the virus (50). Many reasons conspire to make the L452R mutation highly contagious, making it challenging to prevent viruses carrying the L452R mutation.

#### 2.3.6 P681H/R

The P681H/R mutation is located at the junction of the S1/S2 domain of the spike protein and at the cleavage site of the Flynn protease, which is a crucial step for the SARS-CoV-2 to invade cells. Experiments have shown that the S1 subunit is responsible for recognizing ACE2 receptors when the SARS-CoV-2 invades host cells, while the S2 subunit completes membrane fusion by inserting fusion peptides into the host cell membrane to enter cells (51–53). It has been suggested that mutations in P681H/R may cause an increase in the cleavage of S proteins by Flynn’s proteases and proteinase-like proteases, thus enhancing infectivity (54). However, P681H was not regarded as the only mutation, and ignored the effects of other combination mutations on fusion and infectivity. In the detection and surveillance report of the new coronavirus variant in Israel, it was pointed out that P681H was not associated with a higher infection rate and prevalence (55). Prerna Arora and other researchers conducted a functional analysis of the S1\S2 site. They found that P681H mutation did not lead to cleavage of the S1/S2 site of the S protein and pointed out that the S1/S2 site mutation had little effect on ACE2 receptor binding (56). Another mutation in the amino acid residue at position 681 is the P681R mutation, which appears in the Delta variant found in India. The results showed that the P681R mutation promoted the cleavage of S protein by TMPRSS2, led to the activation of the S2 subunit, and accelerated the virus invasion of host cells (57 The increased infectiousness mediated by P681R mutation was similar to that of P681H, and the results showed that the cause of increased infectiousness was identical. Ignored the influence of other mutations on infectiousness and made no systematic evaluation and comparison. Bailey Lubinski used amino acid mutation at position 681 as the only variable to evaluate its effect on infectivity and found that the mutation did not significantly affect virus entry, infection, or cell-to-cell transmission (58). Therefore, the mutation of P681H/R does not significantly impact the infectivity of SARS-CoV-2, and only when combined with other mutations can it realize the enhancement of infectivity.

#### 2.3.7 Q498R

**Figure 8:**
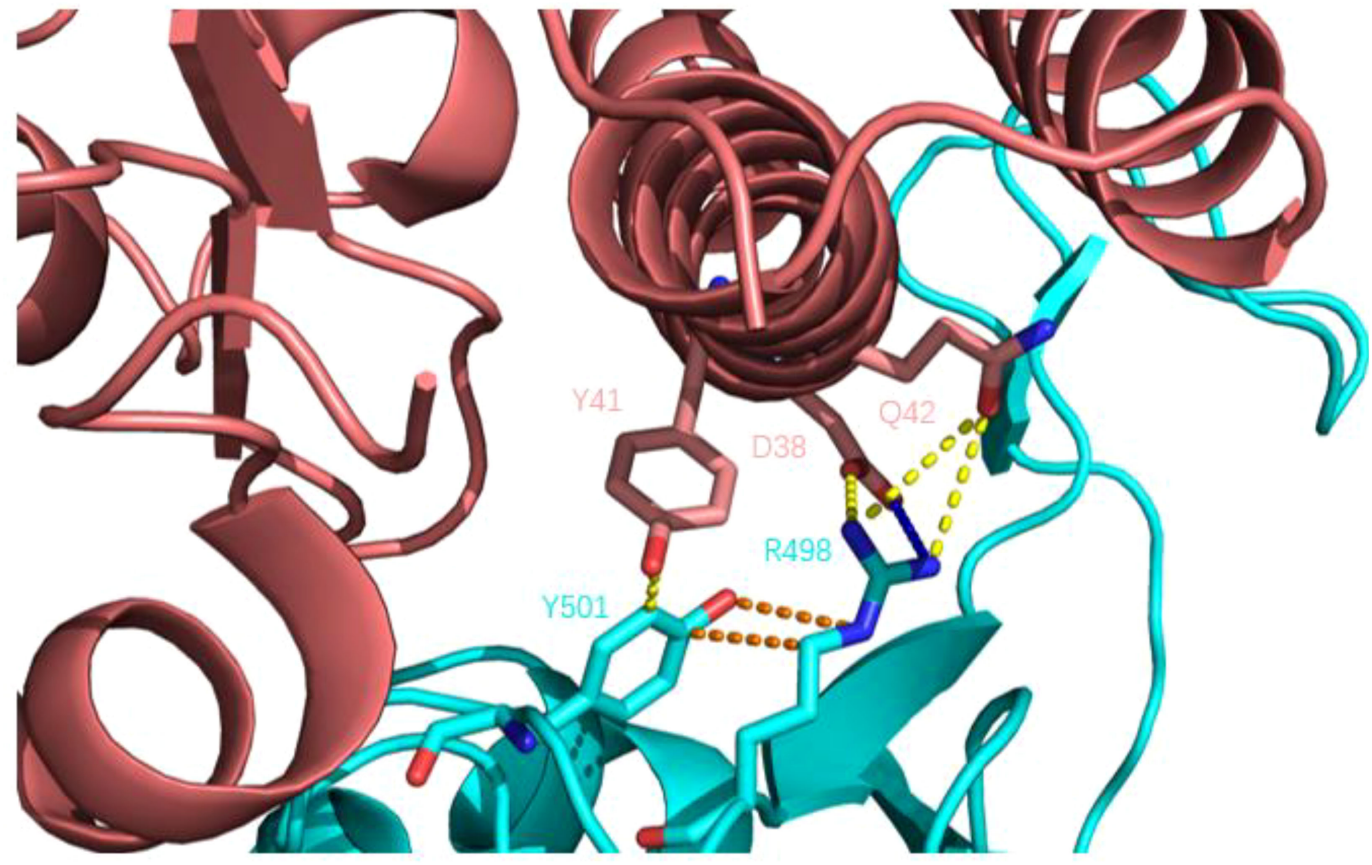
Q498R mutant RBD binding to human ACE2 receptor. Human ACE2 receptors are pink; RBD is blue; yellow dashed lines indicate hydrogen bonds; blue dashed lines show salt Bridges and orange dashed lines show intramolecular interactions. Compared with wild-type novel coronavirus RBD, there is an additional salt-bridge interaction (R498-D38) between the complex structures, which further enhances the stability of the complex, and there is a hydrogen bond interaction between Y501 and R498, further stabilizing the structure of the spike protein.

The Q498R mutation was found in Omicron mutant strains, significantly enhancing its affinity to ACE2 receptors. Some researchers used molecular docking technology to assess the impact of mutations on ACE2 receptor affinity and found that Q498R had the highest docking score with ACE2, even surpassing the N501Y mutation. A high docking score meant that Q498R had a high affinity for ACE2 (59). Subsequently, some researchers used reverse mutation technology to explore the effect of Q498R mutation, reverse mutation Arg498 to Gln498, and found that it significantly reduced the efficiency of Gln498 pseudovirus entering mouse ACE2 receptor cells (60). This also indicates the high affinity of Q498R mutation to the ACE2 receptor. So, what is the mechanism by which Q498R enhances the binding ability of RBD-ACE2? The hot spot residues at the binding interface of RBD-ACE2 were analyzed by molecular dynamics simulation, and the binding affinity hot spot was Q498R (61). Further analysis showed that stronger electrostatic interaction was the main reason for enhancing binding force. Q498R mutation changed from glutamine to positively charged arginine, which enhanced the positive electrostatic potential on the surface of RBD (62, 63). The analysis of amino acid interaction at the binding interface showed that formed a salt bridge between Arg498 and Asp39 (ACE2) (64). As shown in **Figure 8**: In Omicron variant strains, Q498R and N501Y often appeared simultaneously, and the synergistic effect of the two further promoted the infectivity of the novel coronavirus. Mutated Arg498 forms a hydrogen bond with Tyr501, stabilizing the structure of the S protein and further enhancing infectivity (60, 65, 66).

## 3 The impact of mutations on antibody therapy

**Figure 9:**
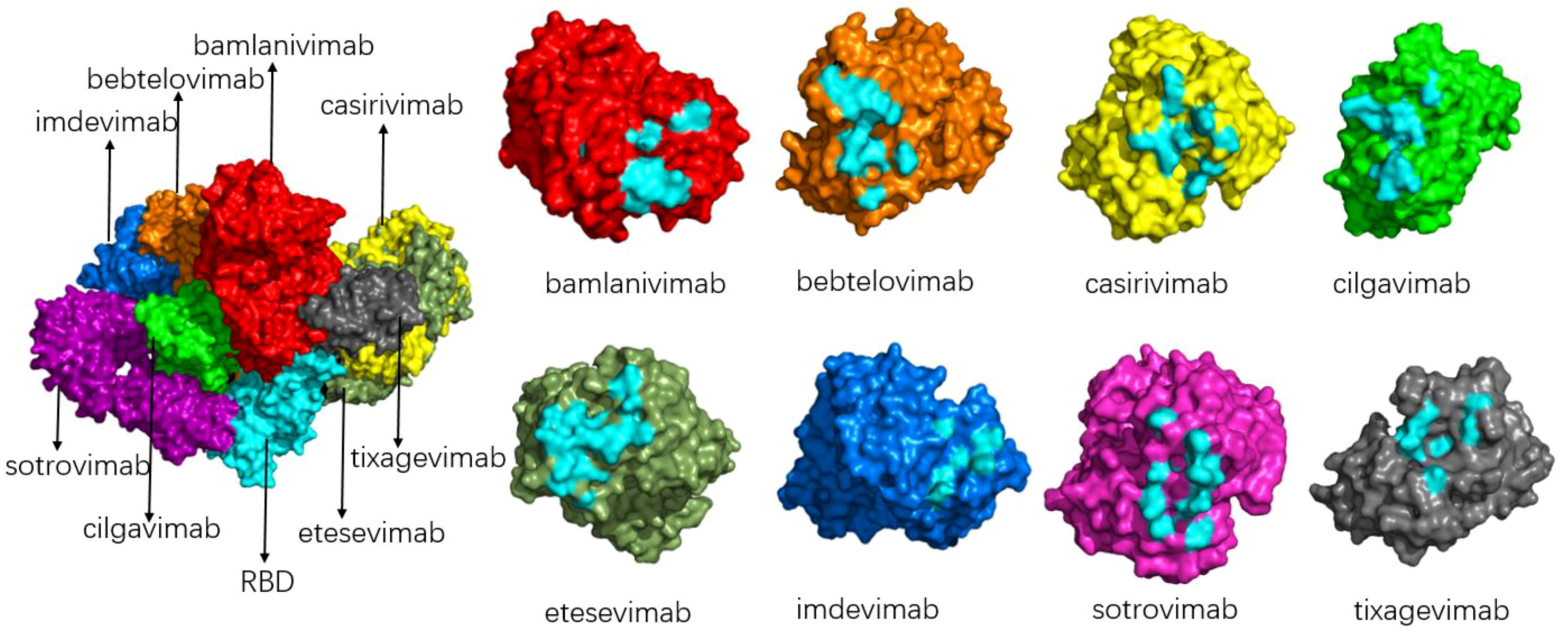
Schematic diagram of the binding of various antibodies to RBD (left) and the binding epitope of each antibody to RBD (right). The composite diagram on the left shows RBD (light blue), bamlanivimab (bright red), bebtelovimab (orange), casirivimab (yellow), cilgavimab (green), etesevimab (grey-green), imdevimab (blue), sotrovimab (magenta), tixagevimab (gray); The neutral epitopes on the right are shown in the same color, and the neutral epitopes are distinguished in light blue.

Because of the epidemic status of COVID-19, several drug development teams and companies around the world have conducted extensive cooperation to jointly research and develop drugs for the prevention and treatment of COVID-19. Monoclonal antibodies with neutralizing activity have the advantages of strong specificity, high safety, clear mechanism of action, ease of mass production, and the ability to prevent and treat COVID-19 simultaneously. They are favored by drug research and development teams. At present, global research and development of COVID-19 antibodies is progressing rapidly. Spike protein is a key component for SARS-CoV-2 to enter cells, and the binding of RBD and ACE2 is the first step of viral invasion (51, 53, 67, 68). Therefore, most of the current neutralizing antibodies target the spike protein of novel coronavirus, aiming to destroy the binding of the RBD-ACE2 receptor (69, 70). (**Figure 9** shows the interaction of a clinically approved monoclonal antibody with the SARS-CoV-2 S protein RBD.) However, with the continuous variation of the spike protein of the novel coronavirus, especially in the RBD region, the therapeutic effect of mAb is affected, and most of the variants have developed resistance to various monoclonal antibodies, as is shown in **Table 2**. The following is a detailed review of the impact of mutations on antibody drugs.

**Table 2:** Neutralizing effect of monoclonal antibody on SARS-CoV-2 variant strains.

WHO	Lineage	Antibody				
		Bamlanivimab+Etesevimab	Casirivimab+Imdevimab	Sotrovimab	Bebtelovimab	Tixagevimab+Cilgavimab
Alpha	B.1.1.7	No change	No change	No change	No change	No change
Beta	B.1.351	Marked reduction	No change	No change	No change	No change
Gamma	P.1	Marked reduction	No change	No change	No change	No change
Delta	B.1.617.2	No change	No change	No change	No change	No change
Omicron	B.1.529	Marked reduction	Marked reduction	No change	No change	Moderate reduction
	XBB.1.5	Marked reduction	Marked reduction	Marked reduction	Marked reduction	Marked reduction
	BA.2	Marked reduction	Moderate reduction	Moderate reduction	No change	Moderate reduction
	BA.5	Marked reduction	Marked reduction	Marked reduction	Moderate reduction	Moderate reduction

### 3.1 Alpha

Alpha came out in September 2020. The number of mutations in Alpha variant strains is small, mainly including △69-70 deletions, N501Y, and P681H, which contain only one mutation, N501Y, in the RBD region; we also mentioned above that N501Y will cause SARS-CoV-2 to have a stronger transmission ability, but will not affect the sensitivity of monoclonal antibodies. The same mutation in S protein P681 does not affect monoclonal antibodies (13). In conclusion, the Alpha variant remains sensitive to monoclonal antibody drugs (18, 71).

### 3.2 Beta and gamma

Beta and Gamma cause the neutralization titers of several monoclonal antibodies to drop significantly, and the mechanism by which they inactivate monoclonal antibodies is similar (18, 72, 73). N501Y, E484K, and K417N mutations were found in Beta variants. Mutations of L18F, K417N/T, E484K, N501Y, and D614G exist in the Gamma variant. As we have learned above, mutations of N501Y and D614G do not affect the neutralization effect of monoclonal antibodies, while L18F is far from the RBD region. In addition, mutations in the amino acid residues K417 and E484, shared by the two variants, are the main reason for the reduced neutralization effectiveness of Beta and Gamma variants against some monoclonal antibodies (74–77). According to the data provided by the US FDA, it was found that the combination of bamlanivimab+etesevimab monoclonal antibodies significantly reduced the neutralizing activity of Beta and Gamma. casirivimab+imdevimab, sotrovimab, bebtelovimab, and tixagevimab+cilgavimab still maintained some neutralizing activity against Beta and Gamma (78). Based on this, the US FDA has restricted the antibody combination of bamlanivimab+etesevimab as a treatment for COVID-19, although the neutralizing efficacy of casirivimab against Beta and Gamma has been reduced. However, the combination of casirivimab and imdevimab still retained some neutralizing activity against Beta and Gamma variants. This also shows that, compared with the single monoclonal antibody therapy, the combination of cocktail antibodies can significantly prevent the escape of the novel coronavirus variant strain, especially the two monoclonal antibodies target different regions of the novel coronavirus RBD, only the amino acids of the two epitopes are mutated at the same time, which may lead to the escape reaction (79). Some researchers studied the effect of E484K mutation on the binding affinity between RBD and several neutralizing nanoantibodies, and found that E484K significantly reduced the affinity between RBD and neutralizing antibodies, mainly due to the electrostatic repulsion interaction caused by the mutation of glutamate to lysine (42).

### 3.3 Delta

The Delta variant is the first to become a global pandemic and is so virulent and widespread that no previous COVID-19 variant has ever been comparable to Delta. The Delta variant does not have mutations such as E484K and K417N/T, but its neutralization can be affected by antibodies (80, 81). The researchers tested the neutralizing effect of several antibodies against the Delta variant. They found that bamlanivimab lost its neutralizing activity against the Delta variant, while etesivimab, casirivimab, and imdevimab remained effective against Delta (82). By isolating a single mutation in the Delta variant, it was found that the L452R mutation was responsible for the loss of neutralizing ability of bamlanivimab (83, 84). Deepali Gupta and other researchers, using molecular dynamics simulation and three-dimensional structural analysis, found that the reason for the reduction of mAb affinity caused by L452R mutation was that the mutation caused structural changes in RBD, and charged plaques appeared near the binding interface with RBD, and the electronegative property was the same as that on the mAb surface, resulting in rejection. (85) Notably, in the molecular dynamics simulation process, it was found that the T478K mutation slightly promoted the binding of RBD to antibodies, but T478K and L452R co-existed and still showed a strong immune escape phenomenon (43, 86). T478K has little effect on the LY-CoV555 monoclonal antibody because T478K is not at the binding interface of RBD-mAb (87). In summary, the L452R mutation is the primary mutation affecting antibody affinity in Delta variant strains. Still, the combination of some mixed antibodies makes immune escape challenging to occur, and most of the neutralizing antibodies retain certain neutralizing activity on Delta.

### 3.4 Omicron and its subspecies

Omicron (B.1.1.529) was first detected in March 2020, and as soon as it was detected, it quickly replaced Delta’s dominance and became popular worldwide (88, 89). Omicron is characterized by high infectivity and viral load, but compared to Delta and other VOCs, Omicron has fewer clinical symptoms and lower hospitalization and mortality rates (90). Omicron is by far the most mutated plant type; 37 different mutations have been found on the S protein alone, which not only leads to the high infectivity of Omicron but also affects the neutralization effect of monoclonal antibodies (91). Even today, the Omicron subspecies is still prevalent all over the world, bringing great disasters to human beings around the world. Omicron mutant strains contain many known mutations in other VOCs, such as K417N, E484A, T478K, etc. These mutations, which will affect the recognition and binding of antibody drugs, have been described above. Almost all the current FDA-approved and EUA clinically approved monoclonal antibodies show no neutralization or low neutralization efficacy in the face of Omicron (92). Only Strovimab and Bebtelovimab maintained the neutralizing activity against Omicron, but the neutralizing titration concentration of Strovimab decreased. The neutralizing epitopes of Strovimab could neutralize Omicron because the neutralizing epitopes of Strovimab were not in the RBM region but in the conserved region of RBD. Mutations in the RBM region had little effect on Strovimab. The decrease in neutralized titration concentration may be due to N440K and G339D mutations in Strovimab binding epitopes, which affected the mAb-RBD interaction (93). Although Strovimab retained some neutralizing activity against Omicron, it significantly reduced its neutralizing efficacy against the Omicron subspecies (BA.2) (94), and the FDA restricted the use of Strovimab due to the trend of the Omicron subspecies pandemic. Bebtelovimab is the only monoclonal antibody to Omicron with a relatively broad spectrum neutralization effect (95). Bebtelovimab targets the conserved region of SARS-CoV-2 S protein RBD, so mutations in the RBD region of the Omicron subspecies have minimal impact on Bebtelovimab (96, 97). Although mutations were also found at the binding interface of Bebtelovimab-RBD complex, such as N440K, G446S, Q498R mutations, etc., these mutations did not affect the interaction between antibody and RBD. On the contrary, Lys440, Ser446, and Arg498 form hydrogen bond interactions with Tyr35, Arg60, and Thr96 of Bebtelovimab to promote the binding of monoclonal antibodies to RBD (98). However, some mutations will also affect the neutralization effect of Bebtelovimab (99). The L452R mutation mentioned above, among others, poses challenges to Bebtelovimab with the continuous renewal of Omicron subspecies and the emergence of new subspecies such as BQ.1, XBB, and XBB1.5 (100, 101). With time, the mutations of the novel coronavirus have become more and more diverse, and new lineages have emerged. As of June 2024, the current VOI are BA.2.86 and JN.1, VUM (Variant Under Monitoring) are KP.2, KP.3, LB.1, and so on. VUM indicates that this variant may require priority attention and monitoring, and whether this variant poses an additional threat to global public health is unknown. KP.2, KP.3, and LB.1 are all subvariants of JN.1. Yu Kaku et al. studied their virology characteristics and found that KP.2 and KP.3 were less infectious than JN.1, but their immune escape ability was enhanced. The infectivity of LB.1 is similar to that of JN.1, and it has a strong immune resistance (102). JN.1 belongs to a subspecies of Omicron and is thought to be a descendant lineage of BA.2.86, with only one change in the spike protein between the two (Leu455Ser), JN.1 showed more than 30 amino acid residues on the spike protein (103). Since JN.1 was first detected in Luxembourg in August 2023, it has spread rapidly worldwide, becoming the fastest-growing COVID-19 variant. On December 19, 2023, the World Health Organization listed it as a “variant of interest” after risk assessment. YuKaku et al. proved that JN.1 was much more infectious than BA.2.86 through a fake virus neutralization test. And showed strong resistance to some antiviral drugs (such as bivalent RNA vaccine, Class I mAb2B04, Class II mAbS309, etc.) (104, 105). All these phenomena indicate that JN.1 is one of the most infectious and immune evasive variants to date.

## 4 Optimization and future design of SARS-CoV-2 antibody drugs

At present, the SARS-CoV-2 is still in the stage of the global pandemic, and dozens of mAb drugs are used to treat the new coronavirus around the world. These approved monoclonal antibodies are advantageous in the early stages of the pandemic. Still, as the novel coronavirus continues to mutate and evolve, there are considerable obstacles to the efficacy of these monoclonal antibodies. Based on this, novel coronavirus antibody drug design still has a long way to go and will develop the future SARS-CoV-2 antibody drugs around the principles of having a broad neutralization spectrum, strong neutralization effect, avoiding antibody-dependent enhancement, and reducing adverse reactions.

### 4.1 Nanobody

Nanobody is a single heavy-chain antibody with a relative molecular mass of about 15KD, about 1/10 of the traditional antibody. It is composed of the CH2 and CH3 constant regions of the heavy chain, hinge, and heavy chain variable regions (106). Compared with traditional monoclonal antibodies, nanobodies have a more extended CDR3 region, can recognize antigen-hidden epitopes, and the physical and chemical properties of nanobodies are relatively stable and can be easily mass-produced (107). At present, antibody treatment for COVID-19 mainly faces two major problems. First, the continuous mutation of the SARS-CoV-2 makes it difficult for some original monoclonal antibodies to be used in newly emerged mutant strains. Second, the infection site of the SARS-CoV-2 is mainly concentrated in the respiratory tract and lungs, and antibodies mainly exist in the blood, which makes it difficult to reach the lesion site to maximize the therapeutic effect. Nanobodies can perfectly solve these two challenges. Nanobodies have a more extended CDR3 region and can access antigenic epitopes inaccessible to conventional antibodies to identify the conserved hidden epitopes of the novel coronavirus and prevent the escape of virus immunity. Due to the characteristics of the nanobody’s low molecular weight, it can achieve inhalation drug delivery and accurately target the lesion.

### 4.2 Multivalent antibody

Multivalent antibody is the polymerization of antibody monomers to form antibody polymers, which can be divided into homologous Multivalent antibodies and heterologous Multivalent antibodies according to the different sources of polymeric monomers. Homologous multivalent antibody forms antibodies that target the same epitope and have stronger neutralizing titers through the polymerization of antibody monomers. Heteromultivalent antibodies conjugate multiple non-overlapping epitopes to obtain antibodies with a broader neutralizing spectrum. At present, many multivalent antibodies are being studied. For example, the pentavalent IgM and bivalent IgA1 antibodies designed by Zhiqiang Ku and other researchers have stronger neutralization ability against SARS-CoV-2. They can also promote the transcell transport of antibodies at the mucosal site to achieve nasal drug delivery (108). In addition, the study of multivalent nanoantibodies is also being carried out (109–111). With the continuous emergence of mutations of the SARS-CoV-2 and the decreasing efficacy of antibodies against different mutant strains, developing bivalent or multivalent antibodies is a vital strategy to solve this problem.

### 4.3 Mixed antibody

A hybrid antibody is a combination of two or more monoclonal antibodies. Mixed antibodies target multiple epitopes of the novel coronavirus, and the synergistic and complementary effects of each monoclonal antibody make the mixed antibodies a better way to treat COVID-19. At present, the combination of two (or more) antibodies is the most effective way to treat SARS-CoV-2, and the existing combination of bamlanivimab and etesevimab, casirivimab and imdevimab, tixagevimab and cilgavimab are all mixed antibodies to treat the SARS-CoV-2. By targeting different epitopes of the SARS-CoV-2 spike protein RBD in a non-competitive manner, the mixed antibodies maintain neutralizing activity against several SARS-CoV-2 variants and have a higher barrier to block immune escape from the SARS-CoV-2 than a single antibody regimen. This is because simultaneous amino acid mutations in both epitope regions are required for immune escape.

## 5 Conclusion

At present, the SARS-CoV-2 is still circulating in the whole region, and the virus mutation is also a continuous process; although the virulence of the new coronavirus has been weakened, it seems more infectious. The mutation of some key amino acids, such as N501Y and E484K, led to the enhancement of viral infectivity. Some amino acid mutations enhance the immune escape ability of the virus, such as L452R, K417N, etc. These mutations were found in the previous mutant strains and have been retained until now, and the combination of some other mutations has led to unpredictable consequences. The immune escape ability of these mutation combinations poses new challenges to the existing antibody drugs. Several of the antibodies initially approved by the FDA to treat SARS-CoV-2 played a crucial role in the early stages of the epidemic, and the continuous evolution of SARS-CoV-2 led the FDA to restrict the use of several monoclonal antibodies, which is unclear in what direction the virus is evolving. In this review, we discuss the molecular mechanisms by which mutations lead to affinity enhancement and immune escape so that we can understand the effects of single-point mutations and roughly understand what the consequences of this mutant strain may be in the face of multiple combinations of mutations. Antibiotic biologics have become a hot spot in global drug research and development in recent years as a targeted therapeutic drug with high specificity, effectiveness, and safety. At present, the most effective means to prevent COVID-19 is vaccination. Still, from a global perspective, due to the backward medical level in some regions, the vaccination rate is very low, and some individuals are not suitable for the COVID-19 vaccine. Even for vaccinated groups, it is not guaranteed that they can effectively prevent the infection, the vaccine will be less protective. We need to build safe and effective COVID-19 treatments in response to these situations. At present, clinical studies on hundreds of novel coronavirus antibody drugs are underway around the world. With the advancement of technology, some new antibodies, such as pre-exposure prophylaxis, long-acting, mixed, and inhalation-administered, have been continuously developed. In the context of the normalization of the COVID-19 epidemic in the future, the new coronavirus antibody will exist for a long time due to its rapid and effective antiviral characteristics, and it can jointly resist the invasion of the new coronavirus with vaccines and small molecule antiviral drugs.
